# Hierarchical Auxetic Mechanical Metamaterials

**DOI:** 10.1038/srep08395

**Published:** 2015-02-11

**Authors:** Ruben Gatt, Luke Mizzi, Joseph I. Azzopardi, Keith M. Azzopardi, Daphne Attard, Aaron Casha, Joseph Briffa, Joseph N. Grima

**Affiliations:** 1 Metamaterials Unit, Faculty of Science, University of Malta, Msida, MSD 2080, Malta; 2 Department of Anatomy, Faculty of Medicine and Surgery, University of Malta, Msida, MSD 2080, Malta; 3 Burns Unit, Mater Dei Hospital, Msida, MSD 2090, Malta; 4 Department of Chemistry, Faculty of Science, University of Malta, Msida, MSD 2080, Malta

## Abstract

Auxetic mechanical metamaterials are engineered systems that exhibit the unusual macroscopic property of a negative Poisson's ratio due to sub-unit structure rather than chemical composition. Although their unique behaviour makes them superior to conventional materials in many practical applications, they are limited in availability. Here, we propose a new class of hierarchical auxetics based on the rotating rigid units mechanism. These systems retain the enhanced properties from having a negative Poisson's ratio with the added benefits of being a hierarchical system. Using simulations on typical hierarchical multi-level rotating squares, we show that, through design, one can control the extent of auxeticity, degree of aperture and size of the different pores in the system. This makes the system more versatile than similar non-hierarchical ones, making them promising candidates for industrial and biomedical applications, such as stents and skin grafts.

Hierarchical materials and structures are a class of systems which are composed of structural elements which themselves have structure^1^. These naturally occurring or man-made systems benefit from significantly enhanced mechanical properties^1^,^2^ such as lightweight high-strength characteristics and an increased resistance to crack propagation^2^. These qualities are needed by biological structures like bones^3^, wood^4^ and insect wings^5^. Hierarchical systems are also utilised in industry and engineering as catalysts^6^, reinforced structures^7^ and sandwich panels^8^.

An equally interesting class of materials and structures are those designed to exhibit auxetic behaviour, i.e. exhibit a negative Poisson's ratio, which means they get wider rather than thinner when uniaxially stretched^9^. Auxetic materials^9^,^10^,^11^,^12^,^13^, like hierarchical systems, also exhibit numerous superior properties, which include synclastic curvature^14^, enhanced indentation resistance^15^ and improved sound damping abilities^16^. Like hierarchical systems, auxetic materials have these properties partly due to their geometric arrangement and the way these deform when subjected to a uniaxial load.

Despite the advantages of auxetic and hierarchical materials, the concept of an auxetic hierarchical system has been poorly studied^17^,^18^,^19^. In particular, despite extensive studies on rotating rigid units and their role in generating auxetic behaviour in various classes of materials such as foams^20^,^21^, silicates^22^,^23^,^24^ and zeolites^25^,^26^, no hierarchical auxetic system based on this concept has as yet been identified. Such hierarchical auxetic systems are expected to provide a new impetus in the design of superior auxetics given the well-known auxetic capabilities of rotating rigid unit mechanisms as well as the superior properties arising from hierarchy.

In this study, we propose and discuss a number of hierarchical auxetics based on rotating rigid units (rotating squares^27^). We study in detail the properties afforded by such systems through the simpler two-level rotating square unit system. Potential applications of these hierarchical auxetics include novel stent design, skin grafting patterns, and other biomedical and industrial devices.

## Hierarchical Rotating Rigid Units

**Figure 1:**
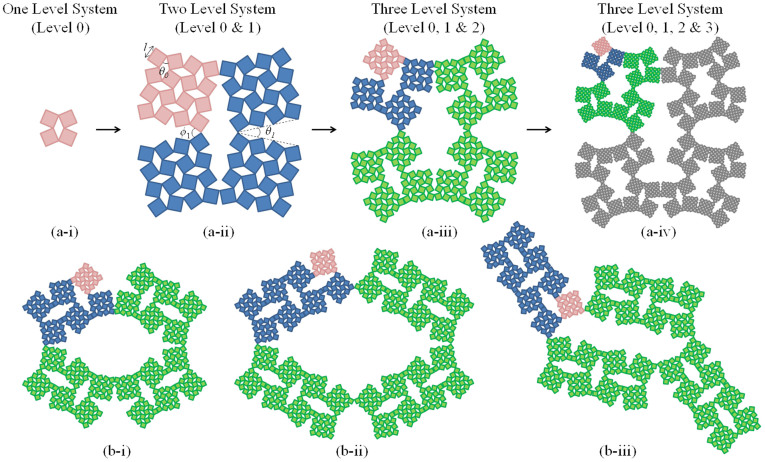
Diagram depicting some different types of hierarchical systems based on the rotating rigid units mechanism. Note that the systems in (a) are all systems were an approximate ‘square' motif is being retained at all levels whilst the systems in (b) are dissimilar hierarchical systems which although being composed of the same level 0 and level 1 units, the sub-units making the level 2 have different shapes, sizes and/or connectivity. Note that in constructing such hierarchical structures care must be taken to avoid overlap, a property which may be partially controlled through changes in the geometric parameters related to the sub-units.

A pictorial description of typical hierarchical systems based on rotating rigid units, proposed in this study, is shown in Figure 1. Note that even for the systems illustrated in Figure 1, made from the classical ‘connected squares' subunits at Level 0, the cases shown here are merely specific cases of much more general hierarchical systems which may be constructed. For example, although the novel hierarchical systems illustrated in Figure 1 consist of multiple levels of two dimensional ‘rotating squares', in general the squares can be replaced by any polygonal shape, e.g. triangles, parallelograms, etc., or even three dimensional polyhedral units. Furthermore, for a given level 0 system, some insight into the versatility of these constructs may be obtained from Figure 1, which highlights how one may build different hierarchical systems either through having different levels of hierarchy (see Figure 1a), or, for a particular level of hierarchy, through the use of different sizes, shapes and connectivities of the sub-levels (see Figure 1b). Obviously different shapes and/or connectivities at any one or more levels can be used, *ad infinitum*, to yield an infinite number of different hierarchical systems which may have different properties and it is beyond the scope of this work to list in detail the full spectrum of auxetic hierarchical systems which may be built in this manner.

Apart from the geometry-based diversity discussed above, for a given geometry, additional versatility may be introduced by having different components and/or levels of the system deforming to different extents. For example referring to the system in Figure 1(a–ii) which shows a system having just levels 0 and 1, it is possible to have some of the level 0 units behaving differently from the others, through, for example, having different constants associated within some of the level 0 units. Alternatively, even if all the level 0 units were to be constructed in the same manner, the structure could still permit significant versatility and tunability simply by using different ‘hinges' between the level 1 units than those used between the level 0 units, i.e., referring to Figure 1(a–ii), having different stiffness constants for the *θ*_0_- and *ϕ*_1_-hinges, a scenario which will be investigated in the present work. Obviously, for any given geometry, there are many such variations that may be introduced, which could have an effect on the overall properties of the system.

It should be also emphasised that when constructing Level *n* from a sub-unit at Level *n*-1, the actual effective shape of system at Level *n* is determined from the exact locations where the different sub-units connect rather than the shape of the sub-unit itself. Thus, for example (as described in more detail in the Supplementary Information), although the sub-units used to build the Level 1 in the systems in Fig. 1a are of a ‘square' shape (they are 4 × 4 connected squares), the connection point between the different sub-units are not at the corners of such ‘squares' as such locations would not correspond to a location of a solid portion. Instead, these sub-units are connected from vertices which are nearest of these corners, with the net effect that the connectivity at Level 1 is best described by the equivalent of a Type I*β* ‘rotating parallelograms' system^28^, or in some cases, the Type I connected rectangles, depending on the angles between the Level 1 sub-units.

## Results and Discussion

To illustrate some of the properties and versatility of the proposed hierarchical structures, simulations were performed on the simple two-level system illustrated in Figure 1(a-ii), where the level 1 and level 0 units were allowed to have different stiffness constants. The hinges between the level 0 squares (angle *θ*_0_), were assigned stiffnesses of 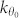 whilst the hinges between the level 1 squares (angle *ϕ*_1_) were given stiffnesses of 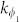. It is important to note that due to the geometry of the system, referring to Figure 1 (a–ii), the level 1 stiffness constant was defined for angle *ϕ*_1_, which is related to *θ*_1_, the angle between through *ϕ* = *θ*_1_ + *θ*_0_. Three different cases were considered here, namely: Case I, where the *ϕ*_1_-hinges were set to be much stiffer than the *θ*_0_-hinges; Case II, where the stiffnesses were set to the exact opposite of those in Case I, and finally Case III, where all the *θ*_0_- and *ϕ*_1_-hinges were given low stiffness constants, i.e. both hinges were soft.

**Figure 2:**
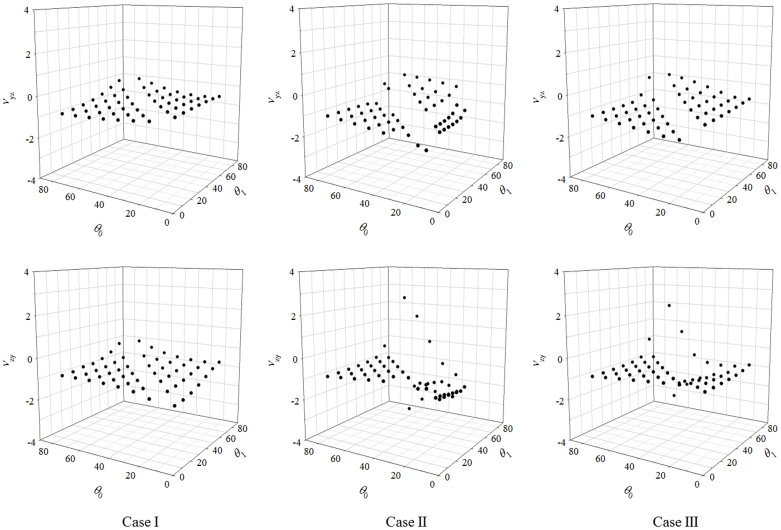
Graphs showing the Poisson's ratios in *ν_yz_* and *ν_zy_* planes for hierarchical structures with different *θ_0_* values. The missing values for *θ_0_ = θ_1_* in both sets were due to unattainable geometries which resulted from mathematical constraints.

Plots showing the on-axis Poisson's ratios *ν*_yz_ and *ν*_zy_ obtained from these systems are shown in Figure 2. These plots show that all systems exhibit auxetic behaviour the magnitude of which is dependent on the geometry of the system and the stiffness constants associated with the *θ*_0_*-* and *ϕ*_1_*-* hinges. The change in angles that occur upon the application of on-axis uniaxial stress help us understand the deformations taking place upon loading (see Supplementary Information). This change in angle clearly suggests that the different sized pores can open up to various extents. The results highlight the versatility of these systems. For example, although the hierarchical structures considered here are based on a rotating squares geometry, a system which has a hallmark isotropic Poisson's ratio of -1, most cases tested here yield different values with a wide range of negative Poisson's ratios. Furthermore, the Poisson's ratio is now direction-dependent with the values for the *ν*_12_ and *ν*_21_ directions differing significantly for the same structures.

**Figure 3:**
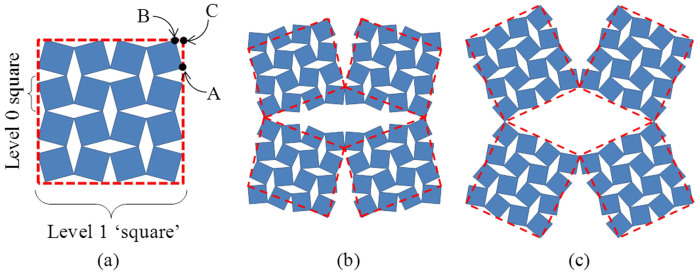
(a) Diagram showing the different points from which the Level 1 unit could be connected to other units (b) a two-level system with an acting rotating unit with a parallelogramic shape and (c) with a rectangular shape.

The simulation of the various systems upon uniaxial loading predict deformation patterns which are highly dependent on the relative stiffness of the different hinges. In Case II systems which have stiff *θ*_0_*-* and soft *ϕ*_1_-hinges behave in such a way that the level 0 geometry does not deform. This means that there are no deformations of the constituent squares themselves or the angles *θ*_0_ between them, as expected, whilst all the deformations occurred at the *ϕ*_1_*-*hinges at level 1. In fact, this behaviour suggests that such systems can be treated as a uni-level non-hierarchical system where the unit which is effectively rotating is the quadrilateral formed by joining the corners containing the *ϕ*_1_-hinges. Here it should be emphasised that these quadrilaterals are not necessarily squares and in this specific case have the general form of a Type I*β* rotating parallelograms or Type I rotating rectangles connectivities, systems which are well known for their non -1 Poisson's ratio properties^28^. This hypothesis is corroborated by the fact that simulated Poisson's ratios for all the Case II systems were found to correspond roughly with those calculated directly using the analytical models^28^ derived for these systems (see Supplementary Information). Here it should be also emphasised that the way the different level 0 units are connected to form the structure at level 1 is critical in determining the overall shape and mechanical properties obtained. In fact, different connectivities, resulting in potentially different properties, could be achieved by varying the manner in which the different level 0 systems are connected together. For example, in this case, as shown in Figure 3b, if the different level 0 systems meet at Point A, something which occurs when *θ*_0_ > *θ*_1_, the ‘effective' rotating unit, would be a Type I*β* parallelogram, whilst if they meet at Point B (see Figure 3c), an effect which occurs when *θ*_0_ < *θ*_1_, the ‘effective' rotating unit would be a Type I rectangle. As evident from the plots in Figure 2, this change is the main the cause for the sudden shift in the mechanical properties of the systems.

For the Case III systems which have soft *θ*_0_*-* and *ϕ*_1_-hinges, the behaviour is such that in all cases the individual squares within the level 0 retain their shape and size, as expected, whilst there were deformations in the angles (see ANIM01.gif). It was observed that the level 1 deformations associated with the *ϕ*_1_-hinges were much more significant than those of the *θ*_0_-hinges at level 0 (see Supplementary Information). This is since much more work would be required to achieve the same extent of uniaxial strain from the level 0 geometry when compared to level 1 geometry. As a result, the overall Poisson's ratio properties of these systems were found to be similar to the Case II systems discussed above.

Case I systems, which have soft *θ*_0_*-*hinges and stiff *ϕ*_1_-hinges, were found to be very rigid and difficult to deform (see Supplementary Information). This may appear surprising given the low stiffness constants of the *θ*_0_-hinges which may suggest easy deformation. However, for the particular systems studied here, the level 0 deformations cannot occur independently from the level 1 deformations. This is because the level 0 squares that are connected to other level 0 squares from adjacent level 1 units can only rotate if there is a change in the stiff *ϕ*_1_ angles. This means that level 0 squares are effectively ‘clamped'.

The main significance of this work goes beyond the results reported above. The concept presented here could be employed in a much wider variety of systems with a range of mechanical properties and applications. One of the more interesting features of these systems is that they could be engineered to have a variable pore size and/or shape (Figure 1). Extent of pore opening depends on the design of the system and, for a given system, the pore sizes would be dependent on the angles between the different units which could be fine-tuned through the use of an externally applied strain. Pore size variability could be very useful in the design and manufacture of smart filters and related systems.

The properties shown here are scale-independent, meaning that the concept of using hierarchical systems based on rotating rigid units can be employed at any length scale ranging from the nano-scale to the macro-scale. At the macro-scale, the auxetic hierarchical system proposed have applications in the construction, manufacturing and transportation industry due to the inherent low-weight associated with hierarchical systems. As shown in the Supplementary Information, a two-level hierarchical system has its density drastically reduced by increasing the values of *θ*_0_. Having a highly auxetic low-weight structure, such as the one presented here has its benefits in applications where a low weight is required along with auxetic features, such as in the manufacture of curved panels for use in aerospace or marine applications due to the suitability of auxetics to form doubly curved surfaces^29^.

At the nano-scale, auxetic hierarchical nano-materials based on the motifs presented here are expected to have their own specific niche of applications, for example, as catalyst supports, where different catalysts controlling different reactions could be placed within specific pores of the material. This function can be achieved since the angles between the sub-units at the different levels of the system, and hence the respective pore sizes, open up to different extents upon application of a strain (see plots in the Supplementary Information).

There are also a number of potential applications in the biomedical field. For example, Alderson et al. had proposed the concept of an auxetic ‘smart' bandage where the degree of swelling would change the rate and extent of medication release^30^. Added tunabilty can be imparted to these smart bandages as a direct consequence of the presence of different pore types which may be made to contain different classes of medications (e.g pain relievers, anti-bacterial agents or anti-swelling agents) that are released at different extents, according to the degree of swelling.

**Figure 4:**
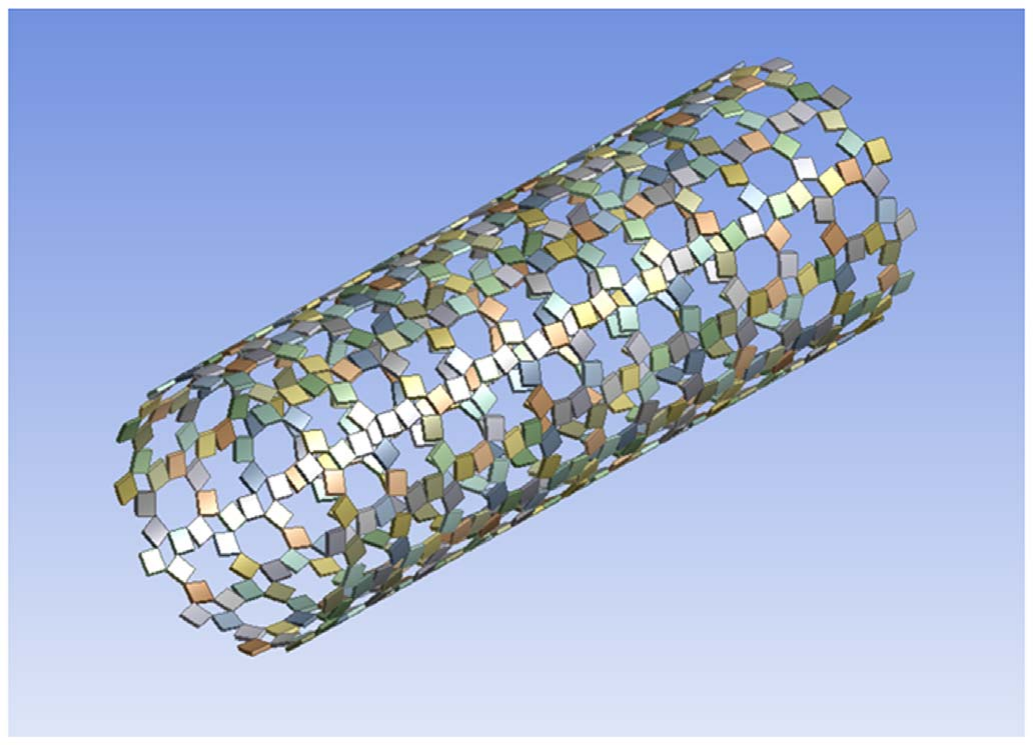
Stent based on a two level hierarchical rotating square geometry.

This concept could also be applied to the design of smart auxetic stents which could be made to exhibit superior properties when compared to existent designs (see Figure 4). Stents based on the rotating square model^27^, i.e. the equivalent of the level 0 system (Figure 1, a–i), have already been proposed for use as oesophageal stents^31^. New stents based on the concepts proposed here, could be engineered to make use of the more flexible levels (in this case Level 1) during the ‘blowing up' process, without affecting the smaller pores which could be used for drug release in an extended and controlled manner. Furthermore, as discussed above and in the Supplementary Information, the hierarchical design significantly reduces the actual surface area of the solid portion with the obvious, but most important result being that one could reduce inflammation occurring due to contact with a foreign body, which is one of the most problematic side effects of stent use, is expected to decrease. Another possible application in the medical field is to make superior skin grafts. Skin grafts usually have a large number of perforations in order to reduce the chances of blood clots and fluid collection under the grafts. The hierarchical systems discussed here, with their lower surface coverage (see above and Supplementary information) are especially well suited for this purpose due to the high number of pores as well as for their propensity to open up these pores upon application of a strain (due to their negative Poisson's ratio), thus relieving the pressure on the swelling area in a more effective manner.

The limitation of the work proposed here is that it is based on a model. For example, the systems used in the simulations were meant to represent ideal, defect-free systems which, for example, at level 0 are made up of perfectly rigid squares of equal shape and size. Such idealised scenarios are difficult to achieve in real applications. The systems being proposed here could be engineered in a slightly different manner, for example, through the use of perforations as detailed elsewhere^32^,^33^. From an implementation point of view, this method of producing such structures could be particularly suitable for the medical applications mentioned above where a sheet of solid material (e.g. the skin in the case of skin grafts) is perforated in an appropriate manner to create a structure that mimics the designs proposed here. Obviously, such experiment-based work would provide the definite proof regarding the true potential of these systems in the various proposed practical applications.

In this work we have proposed a novel auxetic hierarchical system based on the rotating rigid unit mechanism. It was shown that these systems exhibit a wide range of properties which include auxetic behaviour as well as the ability to have different sized pores that can open to various extents. Their size variability is indicated by the changes in the angles between units which is dependent on the relative stiffness of the different hinges in the system. Given the versatility as well as range of applications of the systems proposed here, we envisage that this work will stimulate further research that can lead to the manufacture and commercialisation of auxetic hierarchical systems.

## Methods

### Modelling of hierarchical systems made from squares connected together through hinges

For these simulations, the model structures were constructed using the Materials Studio 6.0 modeling software (distributed by Accelrys Inc.), as planar ball and spring systems described by a harmonic potential as discussed in Grima *et al.*^34^. Referring to Figure 1(a-ii), a series of hierarchical rotating square systems with their respective energy expressions were constructed, where, *θ*_1_, the angle between the level 1 sub-units, defined through the value of *ϕ*_1_*,* calculated accordingly, was given values of *θ*_1_ = 10°, 20°, 30°, 40°, 50°, 60°, 70°, 80°, while, *θ*_0_, the angle between the squares within the level 0, was given values of *θ*_0_ = 10°, 20°, 30°, 40°, 50°, 60°, 70°, 80°, skipping cases where *θ*_1_ = *θ*_0_, due to the fact that these cases are geometrically inadmissible. For all systems constructed, three distinct cases were considered:

- Case I where the *ϕ*_1_-hinges are much more rigid than the soft *θ*_0_-hinges, with 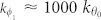;
- Case II where *θ*_0_-hinges are much more rigid than the soft *ϕ*_1_-hinges, with 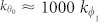;
- Case III where all the *ϕ*_1_- and *θ*_0_-hinges are soft.

In all three cases, the squares were kept as rigid as possible by using the highest values for the stiffness constants to define the internal 90° intra-square angles and side lengths. Full details of the simulations are provided within the Supplementary Information. The mechanical properties including the on-axis Poisson's ratios were calculated using the constant strain method. The deformation mechanisms of these systems were studied by applying incremental uniaxial stresses of up to 5% strain in the *y* and *z*-directions.

## Author Contributions

The concept of hierarchical rotating systems was developed by J.N.G. The simulations were performed by L.M., J.I.A. and K.M.A. J.N.G., R.G. and D.A. wrote the main manuscript text while the figures were prepared by L.M. and K.M.A. A.C. and J.B. contributed to the discussion of the manuscript mainly on the medical aspects of the work involved. The work was supervised by J.N.G. and R.G. All authors reviewed the manuscript before submission.

## Supplementary Material

Supplementary Information ANIM01.gif

Supplementary Information Supplementary Information
