# Supplementary figures and images for: Hierarchical Auxetic Mechanical Metamaterials
Source: Sci Rep. 2015 Feb 11;5:8395. doi: 10.1038/srep08395 (PMC4323639; doi:10.1038/srep08395)

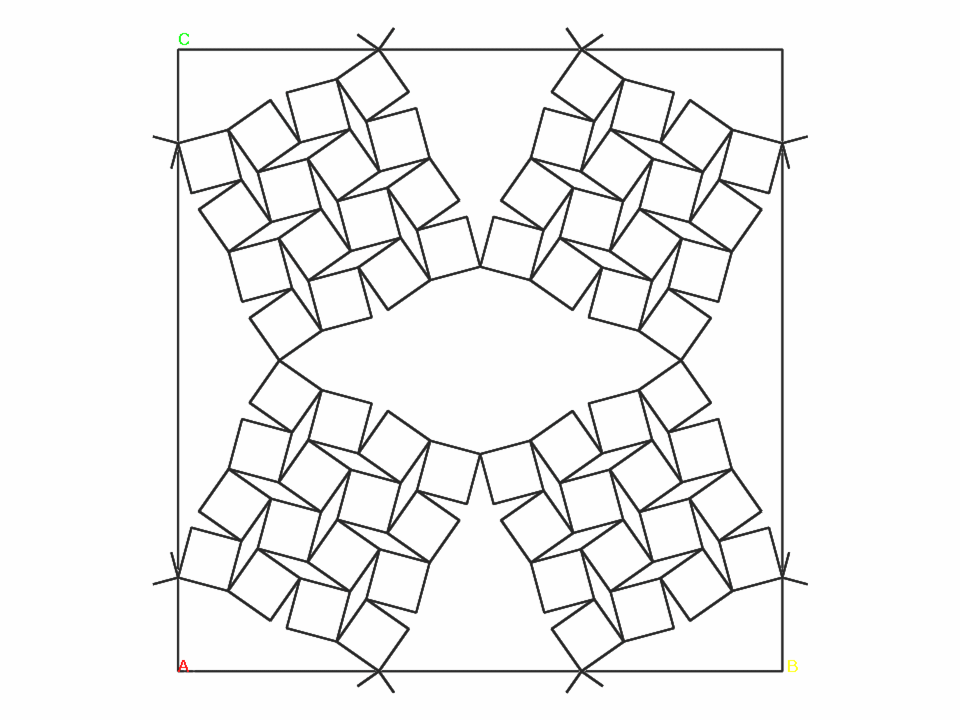

Supplement: Supplementary Information — ANIM01.gif [file srep08395-s1.gif]
