# Supplementary material for: Hierarchical Auxetic Mechanical Metamaterials
Source: Sci Rep. 2015 Feb 11;5:8395. doi: 10.1038/srep08395 (PMC4323639; doi:10.1038/srep08395)
Supplement: Supplementary Information [file srep08395-s2.pdf]

Supplementary Information

Supplementary information for 'Hierarchical Auxetic Mechanical Metamaterials'

by Ruben Gatt[1], Luke Mizzi[1], Joseph Azzopardi[1], Keith M. Azzopardi[1], Daphne Attard[1], Aaron Casha[1,2], Joseph Briffa[3], Joseph N. Grima*[1,4]

[1] Metamaterials Unit, Faculty of Science, University of Malta, Msida, MSD 2080, Malta
[2] Department of Anatomy, Faculty of Medicine and Surgery, University of Malta, Msida, MSD 2080, Malta
[3] Burns Unit, Mater Dei Hospital, Msida, MSD 2090, Malta
[4] Department of Chemistry, Faculty of Science, University of Malta, Msida, MSD 2080, Malta

**Corresponding author: email joseph.grima@um.edu.mt Phone: +356 2340 2840*

---

1. Simulation Methodology

Simulations were performed on various systems based on the idealised model shown in Figure S1 where the different lengths and angles in the system were represented *via* harmonic potentials where the energy terms V_s used to control the lengths were of the form:

$$V_s = \frac{1}{2} k_s (l - l_0)^2$$

where k_s is the stretching force constant, l is the actual length and l_0 is the reference length, whilst the angle bending terms were represented by the harmonic potential V_h given by:

$$V_h = \frac{1}{2} k_h (\theta - \theta_0)^2$$

where k_h is the hinging force constant, θ is the actual angle and θ_0 is the reference angle. Note that the magnitude of the stiffness constants can be used to control the relative stiffness of the different parts in the system. For example, since in our simulations, the squares at level zero were required to behave as much as possible as rigid units, the stiffness constants related to the lengths of the sides of the squares and to the 90° internal angles of the squares were set to a much higher value than the other stiffness constants which related to the parts of the systems which needed to be compliant (e.g. the angles between the different squares). Also, in an attempt to give the systems a depth (hence make them resemble better a real material which would normally be three-dimensional), and at the same time keep the hierarchical motif planar, the hierarchical structures studied here were transformed into three-dimensional periodic systems with multiple layers of 2D hierarchical structures connected together *via* columns emanating from each joint. Through this method of construction, planarity can be ensured by setting the stiffness constants relating to the out-of-plane lengths and angles as high as possible. To simplify the construction and analysis of such constructs, all systems were then modelled *via* the commercially available Materials Studio 6.0 modelling software environment using a methodology based on Grima *et al*. [S1]. This technique, which represents constructs in a manner which is not dissimilar to that used in force-field based molecular modelling techniques, has been validated on a number of related structures [S1, S2] and is considered a suitable *modus operandi* for studying hinging systems such as the hierarchical systems based on squares being investigated here. Note that since periodic boundary conditions were employed, the systems simulated here are a representation of infinite hierarchical structures, i.e. systems which could be considered as one representing a metamaterial.

For these simulations, the stiffness of the angles and lengths of joints and springs making up the system were adjusted according to the cases being investigated where in all cases, the out-of-plane lengths and angles were assigned stiffness constants which correspond to the highest values allowed by the software, i.e., 10^5 Kcal mol^{-1} rad^{-1} and 10^5 Kcal mol^{-1} Å^{-2} respectively. Here it should be noted that the software is coded to use units which are typical to those used in molecular modelling simulations, although they may easily be converted to the appropriate SI units through the appropriate conversion factors as detailed elsewhere [S1]. The Level 0 squares were also represented in a way to behave as rigid as possible (i.e. behave as rigid units) and thus the stiffness constants of the internal angles of these squares and their lengths were also set to the aforementioned values. The rest of the angle bending constants were set according to the cases studied with three main sets of constants being used, namely:

Designation	k_{ϕ_1} (kcal mol^{-1} rad^{-1})	k_{θ_0} (kcal mol^{-1} rad^{-1})
Case I	10^4	10^2
Case II	10^2	10^4
Case III	10^2	10^2

where the maximum stiffness for k_{ϕ_1} and k_{θ_0} was always at least ten times less the value of the stiffness of the internal angle of the Level 0 squares and the stiffness of the beams used to construct them (see Figure S1). This was done in order to ensure that the rotating squares remain as rigid as possible in all cases.

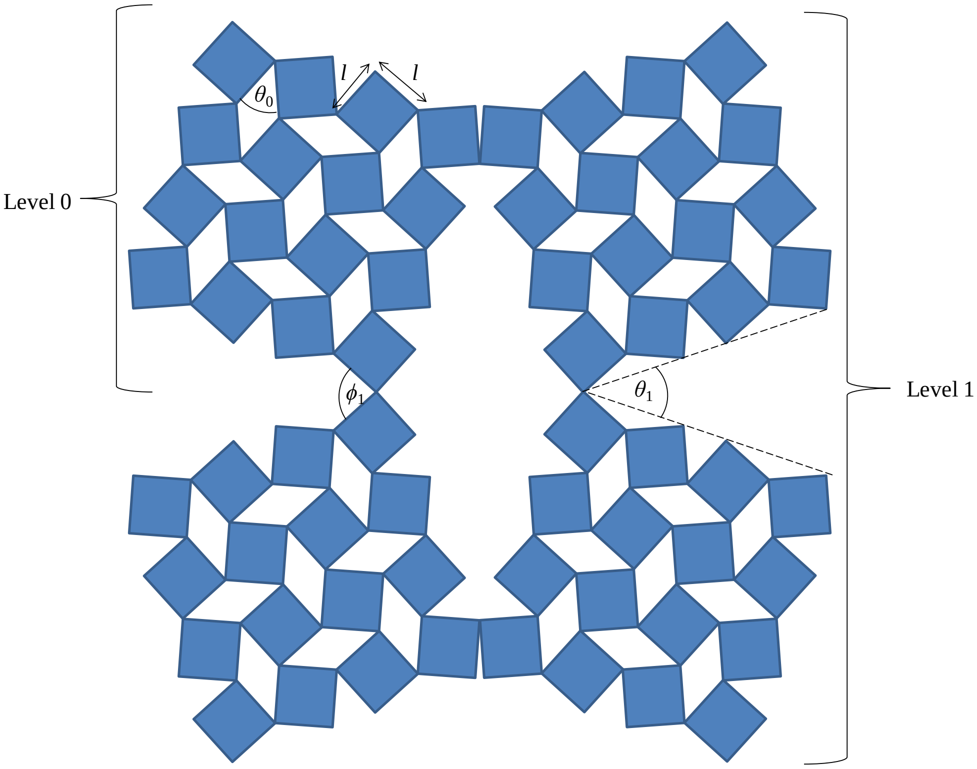

Figure S1: Parameters used to describe a two-level rotating squares hierarchical system.

The energy minimisation was carried out using the SMART minimiser available in Materials Studio 6.0. This minimizer use the Steepest Descent, ABNR (Adopted Basis Newton-Raphson) and the Quasi Newton-Raphson methods in progression to achieve the desired minimum, which in this case were set to an energy value of 0.001 kcal mol^{-1}, a maximum force of 0.5 kcal mol^{-1} Å^{-1}, a maximum stress of 0.5 GPa and a maximum displacement of 0.015 Å. For these simulations, two runs with a maximum of 2000 iterations were used for each structure.

After the minimum energy configuration of the structures was simulated, their mechanical properties were calculated using the constant strain method. In this method, a series of minimizations at different strains (in this case ranging between -0.3% and 0.3%) are performed, with the mechanical properties being derived from the gradient of the resulting stress-strain curves obtained from these minimizations.

2. Analytical Comparison

In the plots below, a comparison between a range of Case II systems and the analytical derivations for Type Iβ parallelograms is shown. Note the Type I rotating rectangles model is merely a special case of this analytical model and thus its mechanical properties can also be predicted using the same equations. In order to compare the mechanical properties of the hierarchical units simulated here with those of the respective non-hierarchical rotating parallelograms/rectangles, the hypothetical lengths (a_p and b_p) and angles (φ_p and θ_p) were obtained by measuring the distances and angles between the points making up the level 1 units as shown in Fig S2.

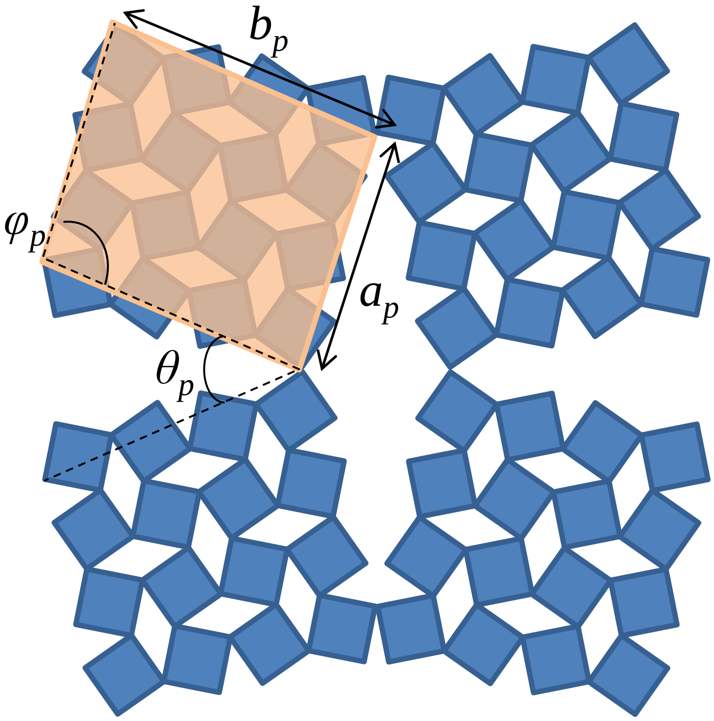

Figure S2: Figure of hierarchical rotating square system showing its non-hierarchical counterpart and the parameters used to describe its rotating unit.

As shown in Fig S3, there is excellent agreement between the simulation and analytical values, except in the case of θ_0 = 50º and θ_1 = 80º. The reason for this discrepancy is the fact that these systems are nearly in their fully opened state and thus their deformation mechanism is not expected to conform with that of the other partially opened systems. In fact, as also shown in Fig 2 of the main paper, the hierarchical rotating square units simulated here with a θ_1 value of 80º show the least auxetic behaviour.

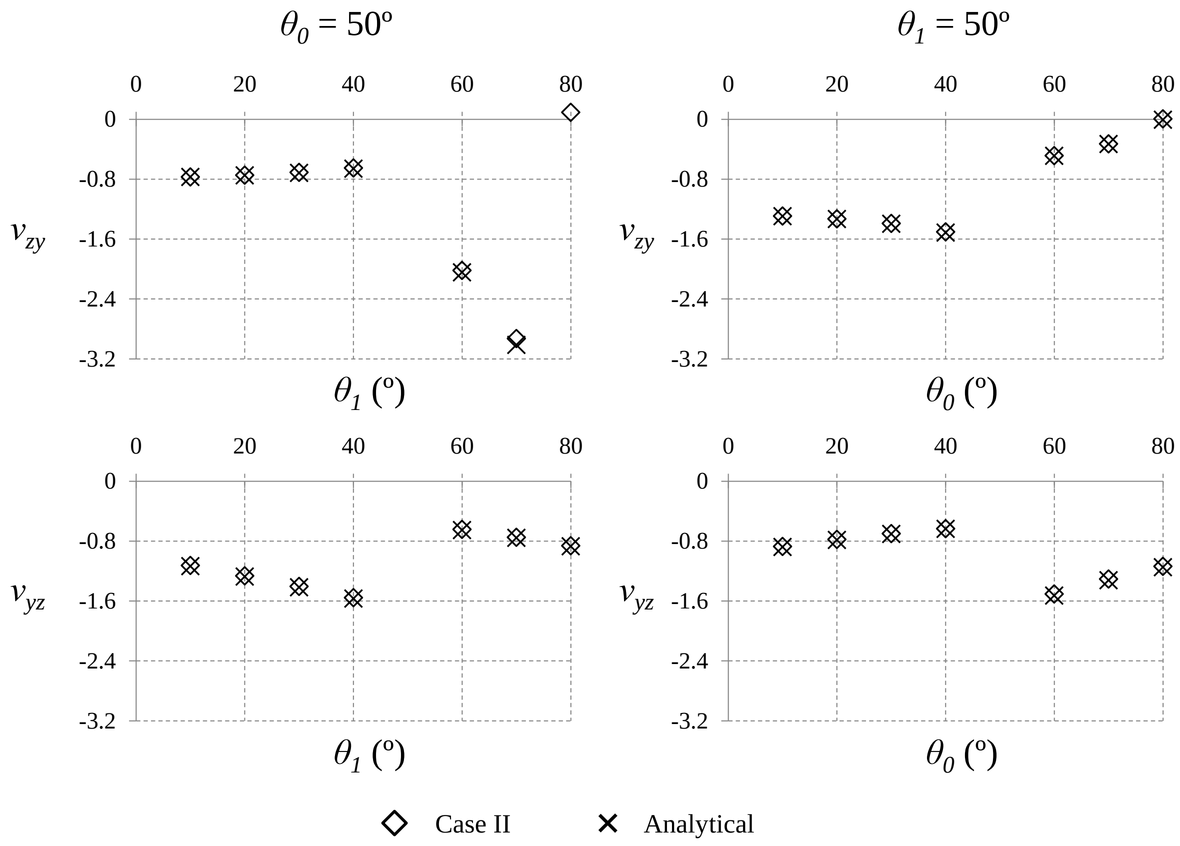

Figure S3: Plots comparing the Poisson's ratio of Case II systems with those obtained using the analytical model for Type I*β* parallelograms (S3). Note that in the case of θ_0 = 50º and θ_1 = 80º, the analytical model reaches an asymptote which is tending to -∞ and thus can no longer accurately predict the Poisson's ratio of the system.

It is also important to keep in mind that the deviation from the expected rotating square behaviour of the level 1 unit is dependent on another factor besides θ_0 and θ_1. As mentioned earlier in the main text, the number of level 0 units also has a prominent effect. As shown in Figure S4 below, the lesser the number of level 0 units, the greater the deviation from the 'ideal' square level 1 unit. However if the number of level 0 units is sufficiently increased, this deviation becomes negligible and the level 1 of the system would effectively have the shape of a square.

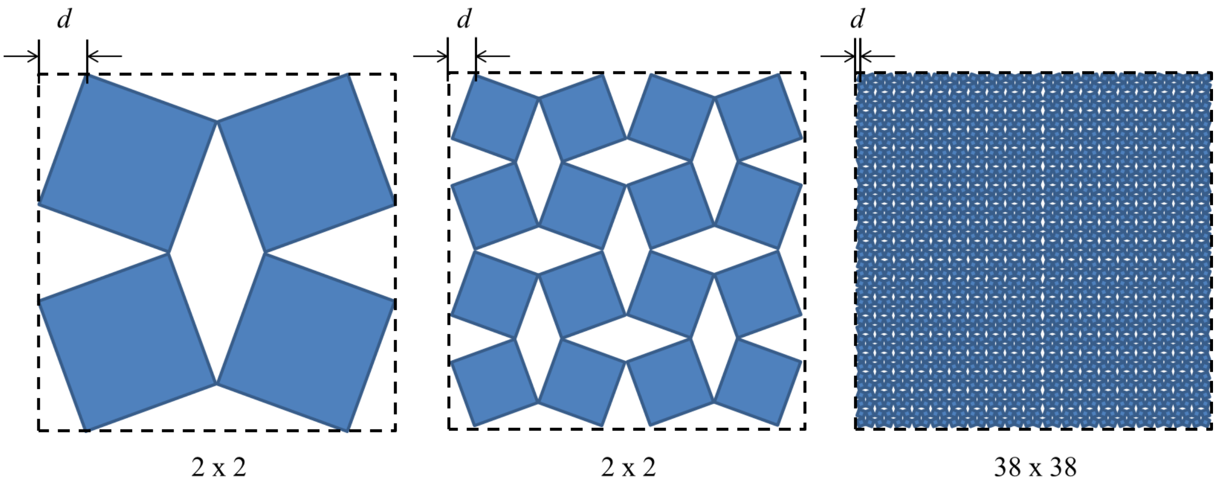

Figure S4: Diagram depicting the variation in the shape of the level 1 unit with increasing number of level 0 units. As evident from the figure, the great the number of level 0 units, the lesser the deviation, *d*.

3. Young's Moduli

The respective Young's Moduli for all hierarchical systems simulated here can be found in the 3D plots shown in Figure S5 below. As one can note, Case I systems exhibit significantly higher Young's Moduli when compared with Case II and Case III systems as a result of the clamping effect mentioned in the main manuscript. Also, systems with θ_1 of 80º show the highest Young's Moduli due to the fact that they are the closest to the fully opened conformation of this system.

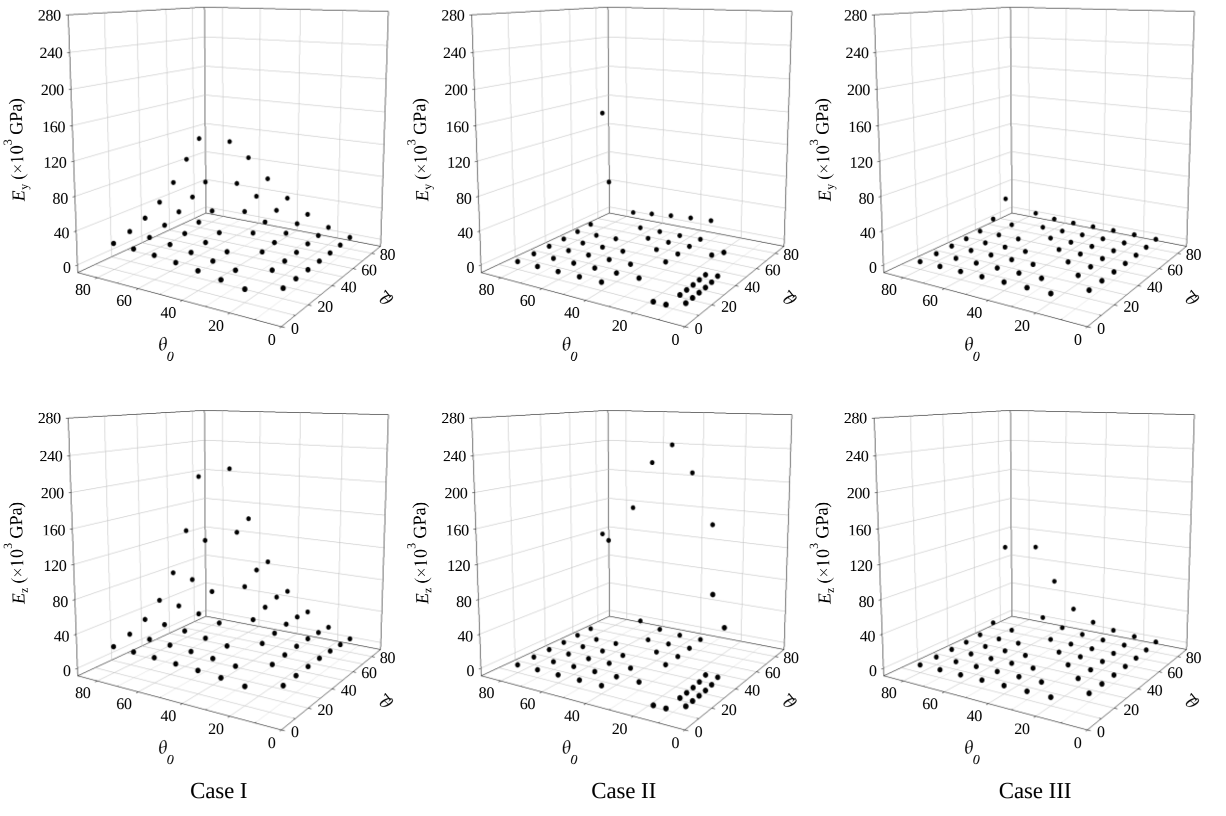

Figure S5: Plots of the variations in the Young's Moduli of various hierarchical rotating unit systems.

4. Density and Surface Coverage Analysis

One particular advantage which hierarchical rotating units enjoy over their non-hierarchical counterparts is a reduced surface coverage to overall area ratio. As shown in the plots in Fig S6, hierarchical systems show significantly less surface coverage, and therefore a lesser density, when compared with non-hierarchical systems. This could be particularly useful in cases where lightweight auxetic systems are required.

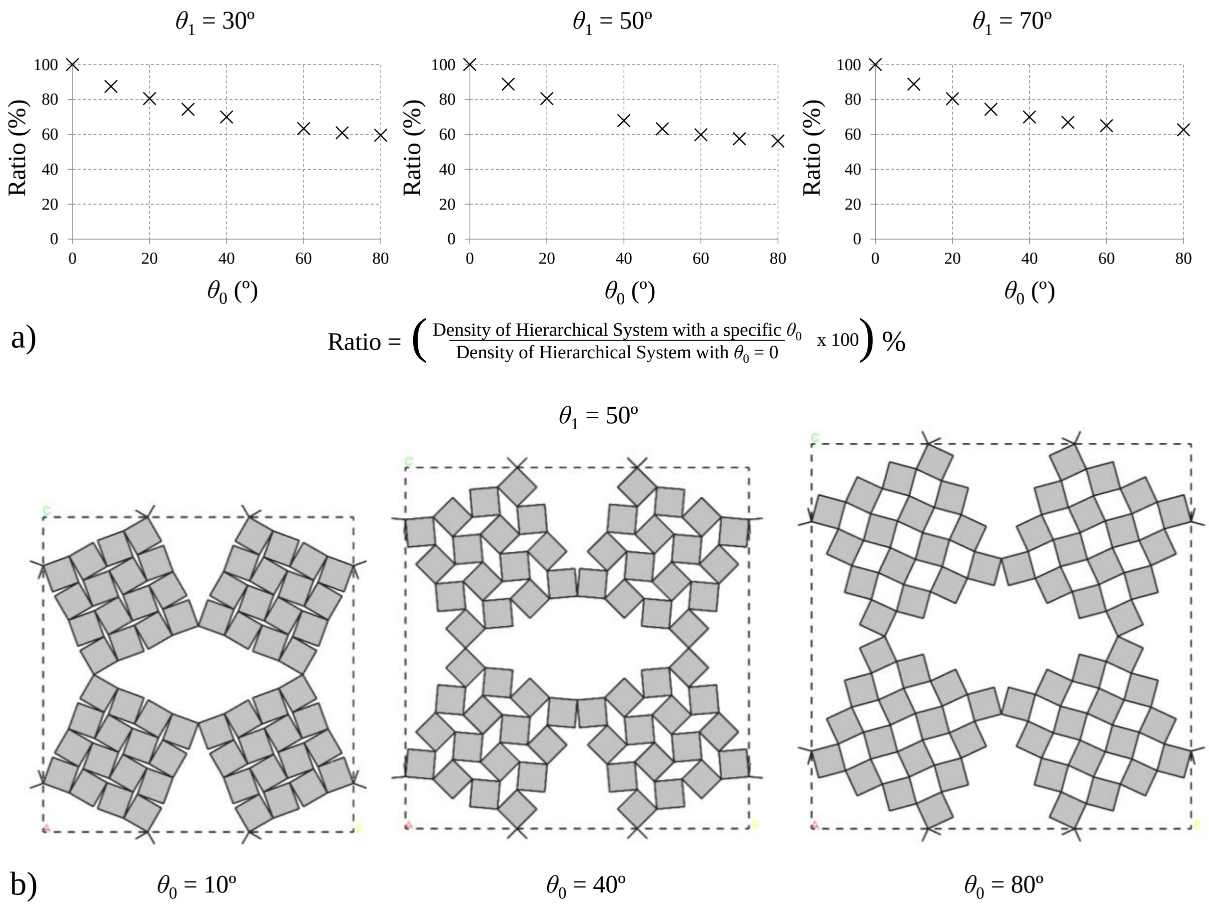

Figure S6: a) Plots showing how variations in θ_0 affect the density of the overall system and b) figures depicting a range of θ_0 values for hierarchical systems with a θ_1 value of 50º. Note that as θ_0, increases, so too does the area of the unitcell, whilst the amount of surface coverage remains unchanged.

5. Deformation on Application of Strain

In an attempt to simulate the deformation profile of these systems upon application of uniaxial strain, further simulations were performed where the systems were subjected to incremental strains of upto 5% in steps of 1% in the Ox_1 and Ox_2 directions. The deformation mechanisms of these systems was then studied in terms of the changes in the angles θ_1 and θ_0 where changes in θ_1 and θ_0 as predicted for the simulation are reported in Figure S3 below. Note that the values reported here are an average of all the θ_1 and θ_0 angles present in each hierarchical structure, along with the standard deviation for each point.

As shown in Fig S7a-b, as expected, the main deformation for Case I systems occurs in the level 0 units while for the Case II and Case II systems it occurs in the level 1 units. Surprisingly, despite being much more stiffer than the θ_0 hinges, in Case I systems, the φ_1 angles still show significant deformation. Coupled with the relatively high Young's moduli observed for these systems (see Figure S5), this further confirms the hypothesis that by making the φ_1 hinges more rigid, one is clamping the system. For the Case II and Case III, the change in angle of the θ_0 hinges is minimal when compared with that of the φ_1 hinges, even in cases when the stiffness of both hinge types is the same, which highlights the higher propensity of these hinges to deform. In all Case II and Case III systems, the standard deviation was extremely small, meaning that all pores are deforming more or less in the same manner.

Case I θ_1 = 50º

Strain in *y*-direction

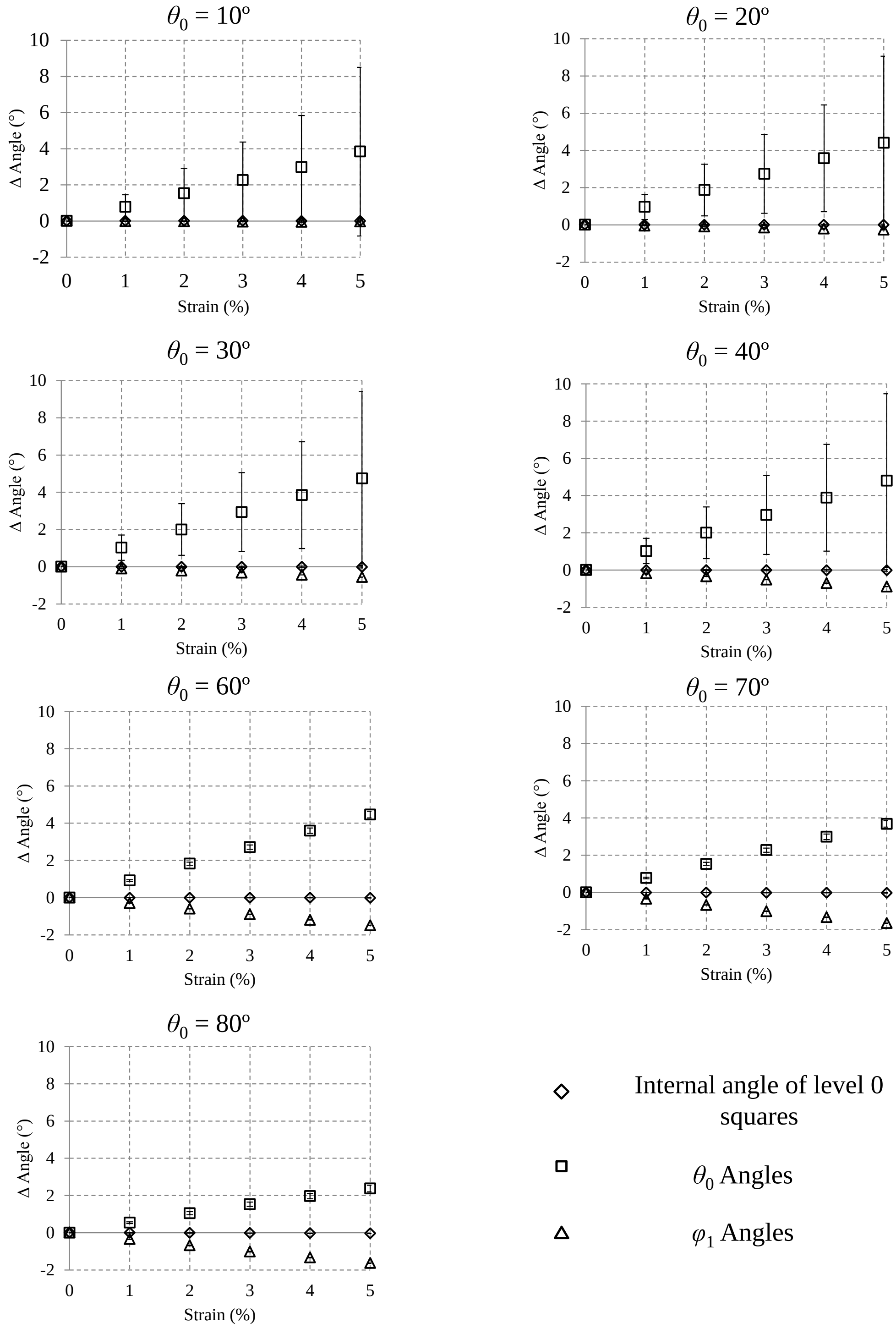

Figure S7a: Plots showing the change in angles of Case I hierarchical rotating square systems for strain in the *y*-direction where θ_1 = 50º and θ_0 = 10º … 80º. The large standard deviations are observed as a result of the restriction place upon the deformation of the entire system by the θ_1 hinges.

Case I θ_1 = 50º
Strain in *z*-direction

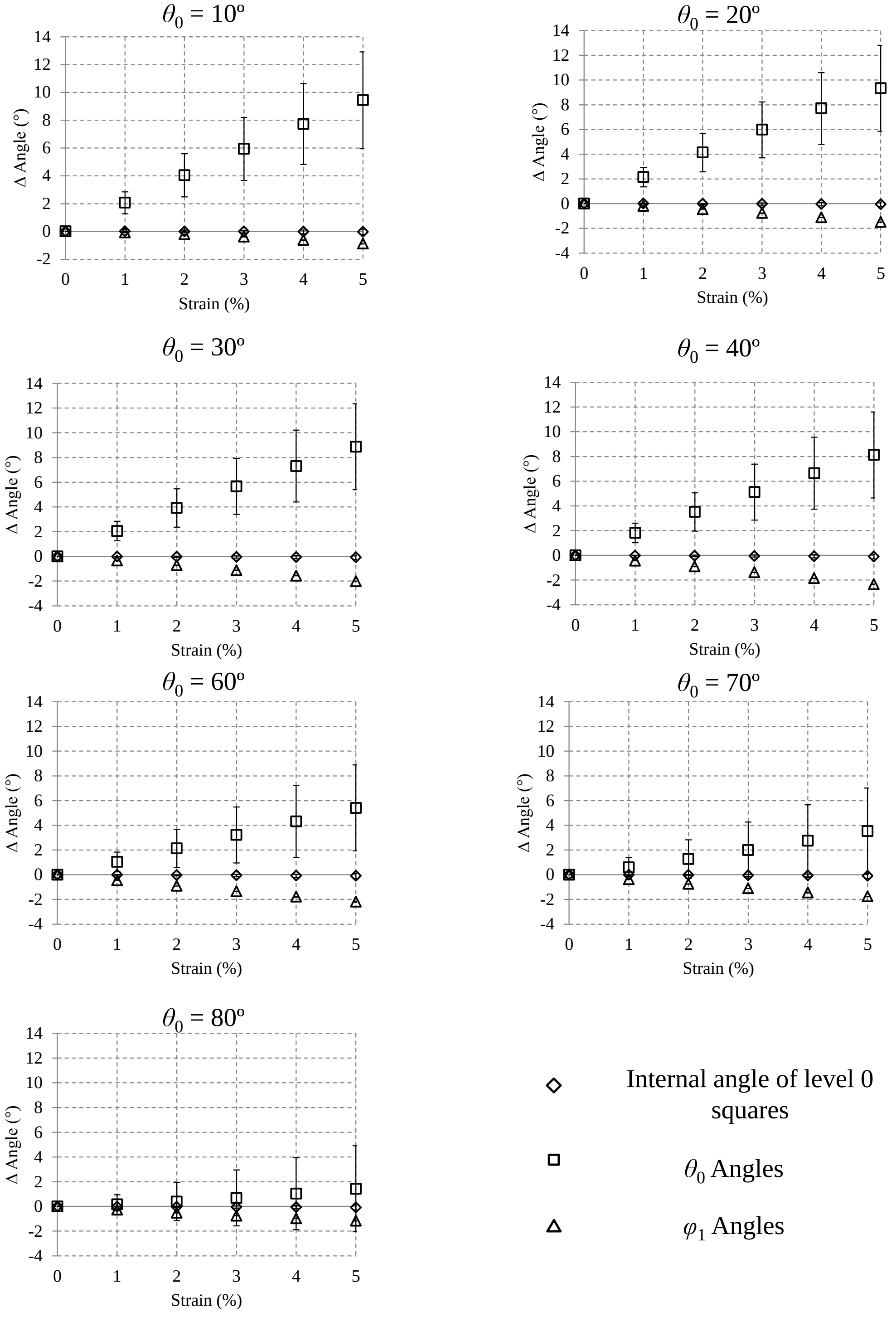

Figure S7b: Plots showing the change in angles of Case I hierarchical rotating square systems for strain in the *z*-direction where θ_1 = 50º and θ_0 = 10º … 80º. The large standard deviations are observed as a result of the restriction place upon the deformation of the entire system by the θ_1 hinges.

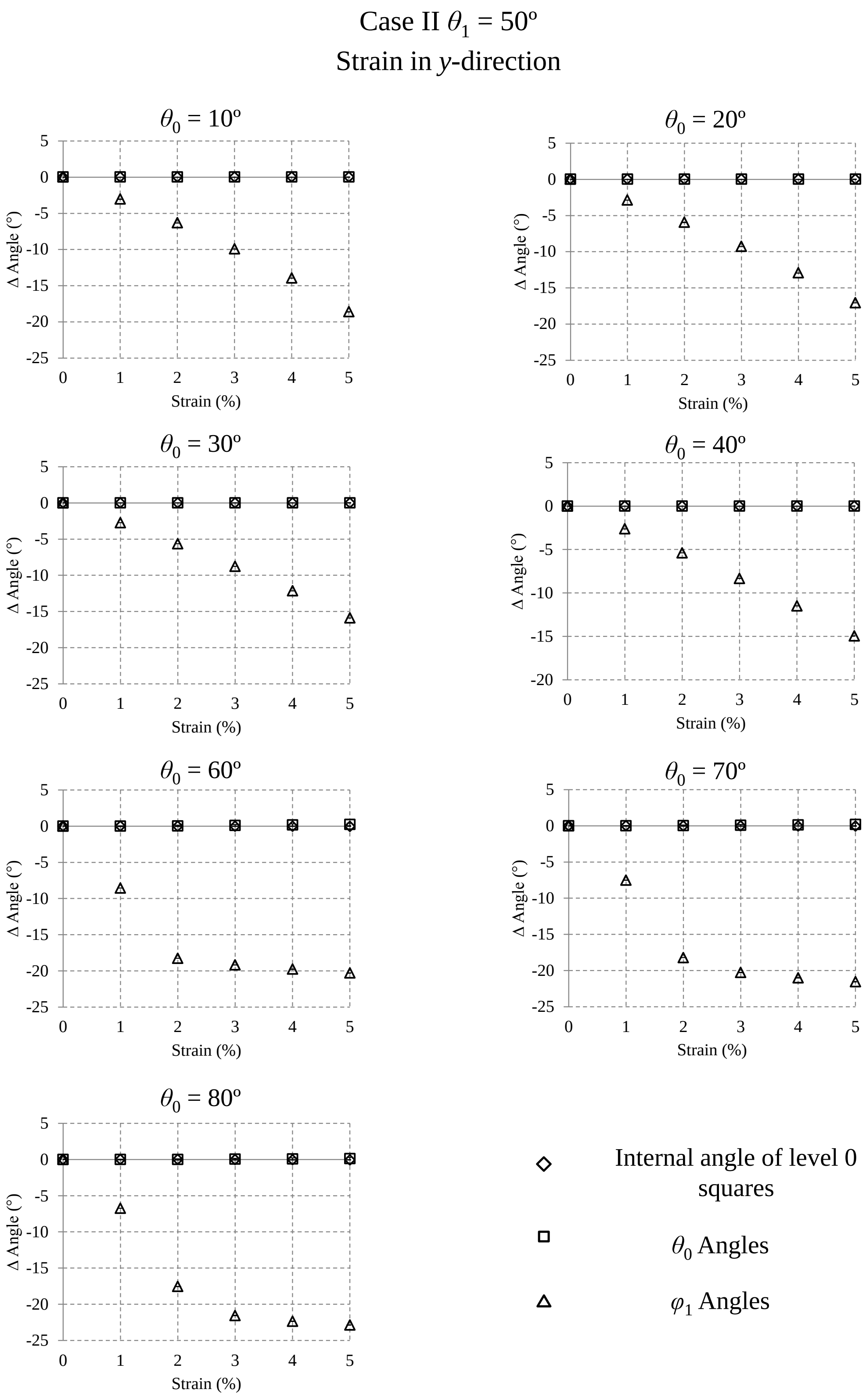

Figure S7c: Plots showing the change in angles of Case II hierarchical rotating square systems for strain in the y-direction where θ_1 = 50º and θ_0 = 10º … 80º.

Case II θ_1 = 50º

Strain in z-direction

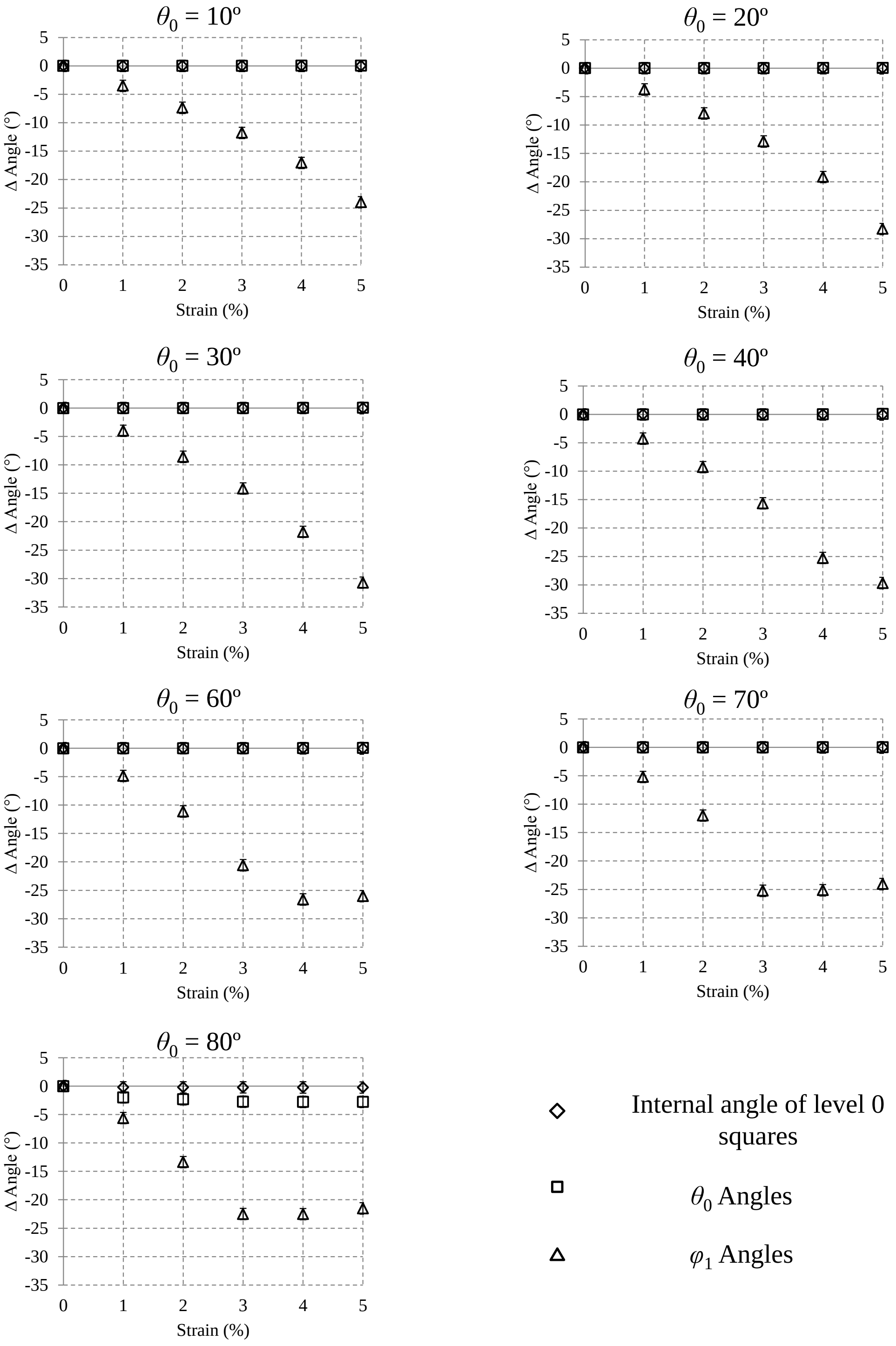

Figure S7d: Plots showing the change in angles of Case II hierarchical rotating square systems for strain in the *z*-direction where θ_1 = 50º and θ_0 = 10º … 80º.

Case III θ_1 = 50º

Strain in *y*-direction

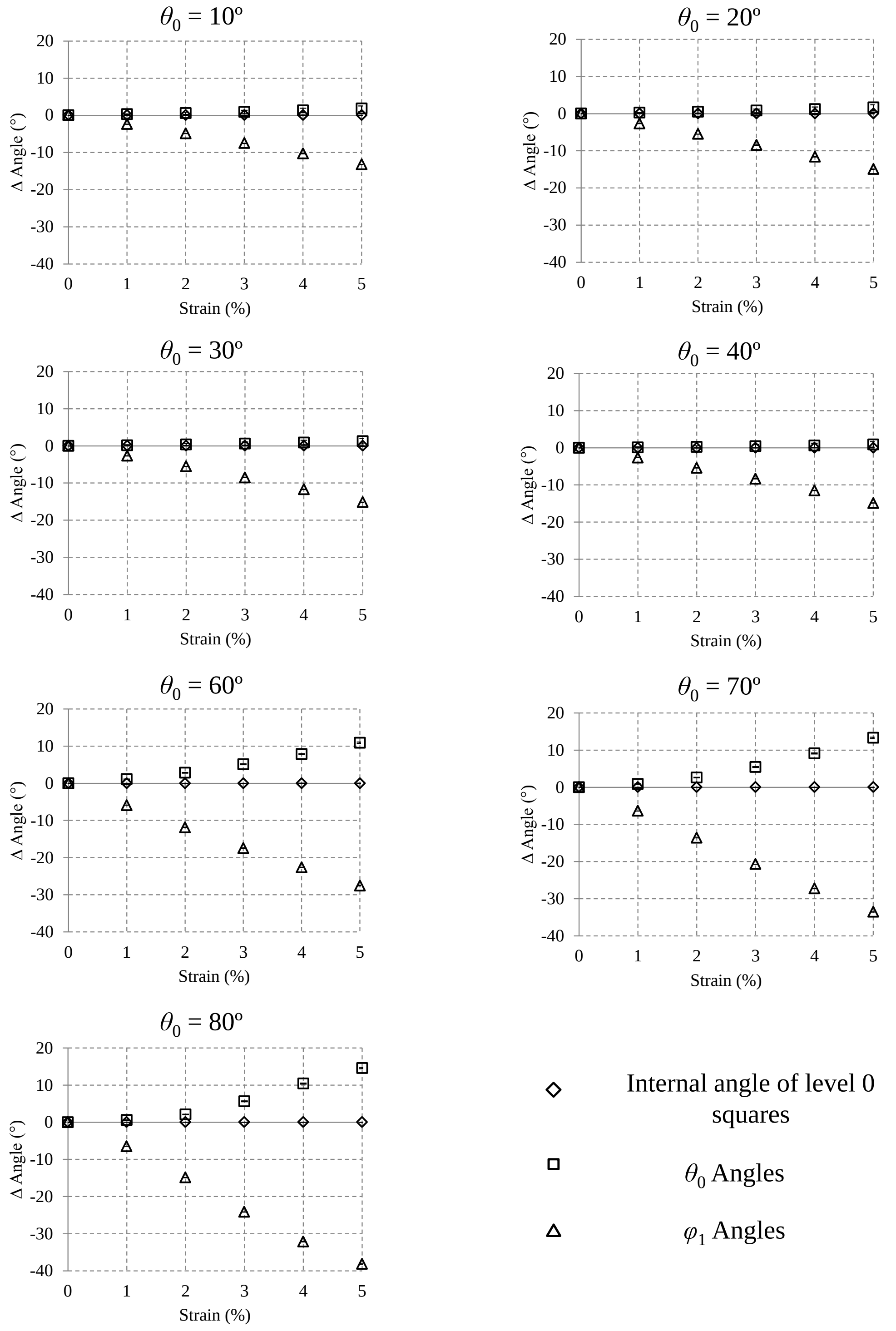

Figure S7e: Plots showing the change in angles of Case III hierarchical rotating square systems for strain in the *y*-direction where θ_1 = 50º and θ_0 = 10º … 80º.

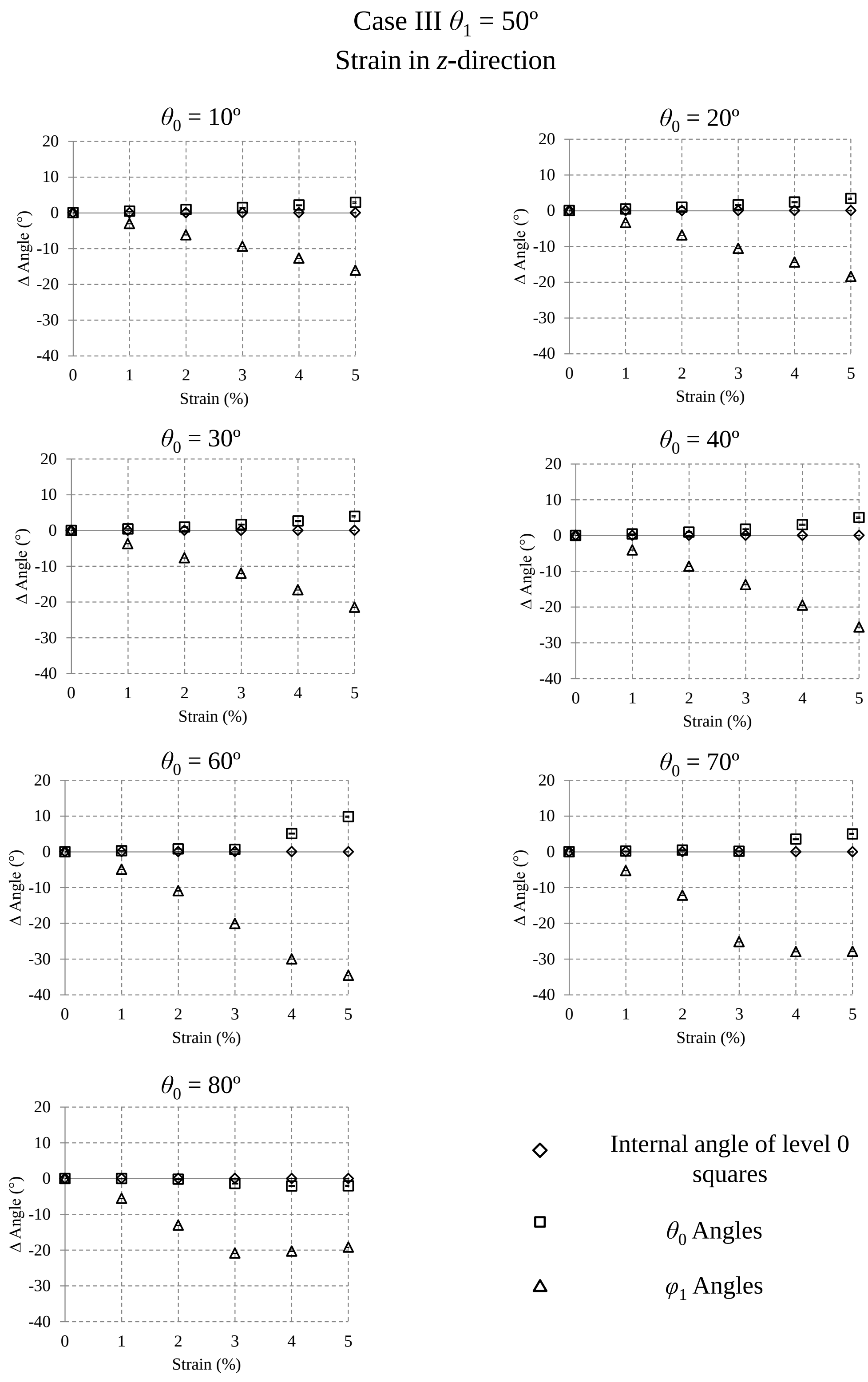

Figure S7f: Plots showing the change in angles of Case III hierarchical rotating square systems for strain in the z-direction where θ_1 = 50º and θ_0 = 10º … 80º.

References:

[S1] Grima, J.N., Gatt, R., Chircop Bray, T.G., Alderson, A., Evans, K.E., Empirical modeling using dummy atoms (EMUDA): an alternative approach for studying "auxetic" structures, *Mol. Simulat*, **31**, 915-924 (2005).

[S2] Grima, J.N., Gatt, R., Alderson, A., Evans, K.E., On the potential of connected stars as auxetic systems, *Mol. Simulat.*, **31**, 925-935 (2005).

[S3] Attard, D., Manicaro, E., Grima, J.N., On rotating rigid parallelograms and their potential for exhibiting auxetic behaviour, *Phys. Status Solidi B*, **246**, 2033-44 (2009).
